# Association between ambient air pollution exposure in pregnant women with antiphospholipid syndrome in Nanjing, China

**DOI:** 10.1007/s11356-023-29937-0

**Published:** 2023-11-01

**Authors:** Bimei Hu, Linjie Xu, Xu Yang, Shiwen Qu, Lan Wu, Yumei Sun, Jun Yan, Yexiao Zhang, Zhaoer Yu, Yixiao Wang, Ruizhe Jia

**Affiliations:** 1 Department of Obstetrics, Women’s Hospital of Nanjing Medical University, Nanjing, 210000 Jiangsu China; 2 Lianyungang Branch of Traditional Chinese Medicine, Jiangsu Union Technical Institute, Lianyungang, 222000 Jiangsu China; 3 State Key Laboratory of Reproductive Medicine, School of Public Health, Nanjing Medical University, Nanjing, 210000 Jiangsu China; 4 Key Laboratory of Modern Toxicology of Ministry of Education, School of Public Health, Nanjing Medical University, Nanjing, 210000 Jiangsu China; 5 Information Center, Women’s Hospital of Nanjing Medical University, Nanjing, 210000 Jiangsu China

**Keywords:** Antiphospholipid syndrome, Atmospheric pollutants, Blood indicators, Birth weight

## Abstract

Antenatal exposure to air pollutants is thought to be associated with a variety of maternal blood markers as well as adverse birth outcomes. However, the dysgenic influence of air pollutants on the antiphospholipid syndrome (APS) in mothers and their pregnancy outcomes remains unclear. In the current study, 371 mother-infant pairs (189 healthy: 182 APS) from Nanjing Maternal and Child Health Hospital as well as air pollutants concentration from their living environment were used to investigate correlations between air pollution with maternal blood indicators and fetal birth weight in the groups of APS and healthy mothers. Generalized linear model was used to evaluate the contributions of air pollutant exposure during pregnancy to the blood indicators variation. The relationships between birth weight with specific air pollutant and blood index were analyzed using ridge regression. Results showed that APS fetal birth weight was significantly impacted by air pollutant exposure during pregnancy, in particular, the birth weight decreased significantly along with increasing fine particulate matter 2.5 (PM_2.5_) and fine particulate matter 10 (PM_10_) exposure concentrations throughout pregnancy. In contrast, birth weight increased significantly with sulfur dioxide (SO_2_) exposure. In addition, APS-related blood indicators comprised of platelet distribution width (PDW), total bilirubin (TBIL), mean platelet volume (MPV), platelet-larger cell ratio (P_LCR), homocysteine (HCY), alkaline phosphatase (ALP), direct bilirubin (DBIL), basophilic granulocyte (BAS), platelet thrombocytocrit (PCT), preprandial glucose levels (OGTT0), monocytes (MON), and monocytes ratio (MON_ratio) were also strongly related with prenatal exposure to PM_2.5_ and PM_10_, in which PDW levels showed most strongly negative impaction on fetal birth weight. Together, we showed that prenatal exposure to air pollutant (PM_2.5_ and PM_10_) may exacerbate the poor birth outcomes of low birth weight by impacting APS maternal blood indicators especially for PDW.

**Supplementary Information:**

The online version contains supplementary material available at 10.1007/s11356-023-29937-0.

## Introduction

Ambient air pollution has long been recognized as a significant factor impacting human physical and mental well-being (Hu et al. 2022; Xue et al. 2020). Prolonged exposure to elevated air pollution levels can give rise to a myriad of health issues, encompassing respiratory diseases (Jerrett 2022), cardiovascular diseases (Hayes et al. 2020), and more, consequently contributing to the global mortality rate. Tragically, air pollution is responsible for over 65 million deaths worldwide annually (Fuller et al. 2022), with vulnerable populations, such as pregnant women and children, bearing a heightened susceptibility to its effects (Klepac et al. 2018; Nobles et al. 2019). Ample evidence points to the potential risk air pollutants pose during pregnancy, leading to adverse complications and outcomes, including gestational diabetes, pre-eclampsia, miscarriage, preterm labor, and low birth weight (Gao et al. 2022; Pedersen et al. 2013; Stieb et al. 2012). However, comprehending the effects of ambient air pollution on maternal and infant health remains a complex undertaking, given the diverse array of air pollutants and their interdependence with maternal fitness and environmental conditions. As a result, a more profound understanding of the relationship between ambient air pollution and pregnancy disorders is imperative, as it can pave the way for more effective eugenics and improved maternal and infant protection measures.

APS is an autoimmune disorder characterized by hypercoagulability, potentially leading to venous and arterial thrombosis, pregnancy loss, and preterm delivery, particularly in severe pre-eclampsia or placental insufficiency patients (Ruiz-Irastorza et al. 2010). This condition can be identified by the presence of at least one antiphospholipid antibody (anti-cardiolipin antibody, anti-β2 glycoprotein-I, or lupus anticoagulant) in pregnant women, alongside abnormalities in a wide range of blood parameters (Miyakis et al. 2006). For example, early pregnancy thrombocytopenia is associated with an elevated risk of preterm delivery in APS-afflicted women (Jin et al. 2022; Uthman et al. 2008), and increased neutrophil activation has been observed in APS patients (Knight et al. 2017; Wirestam et al. 2019). Exposure to ambient air pollutants can also be found to adverse maternal health and pregnancy outcomes by disrupting normal blood indicators. For instance, exposure to PM_2.5_ or certain ambient air pollution components can be found to associate with the blood glucose homeostasis disruption in gestational diabetes patients (Yao et al. 2020), the hemoglobin concentration levels reduction in late maternal pregnancy (Xie et al. 2022), and even the total thyroxine (TT4) levels in offspring (Irizar et al. 2021). Despite ambient air pollutants have been reported to affect maternal blood indicators at different pregnancy stages, there remains limited evidence regarding whether and which maternal blood indicators can be impacted by exposure to ambient air pollutants in APS patients. As such, there is a particularly urgent need to study and clarify the real impacts of ambient air pollutants on maternal health with APS, considering the continuous deterioration of ambient air qualities.

In this research, we recruited a total of 371 mother-infant pairs, with 182 mothers having APS and 189 being healthy controls, from Nanjing Maternal and Child Health Hospital. Throughout the preconception period and pregnancy, we assessed various air pollutants, including particulate matter (PM), nitrogen dioxide (NO_2_), sulfur dioxide (SO_2_), carbon monoxide (CO), and ozone (O_3_). Our investigation focused on examining the impact of air pollutant exposure during the initial 90 days of pregnancy and at different gestational periods on maternal blood indicators. Furthermore, we analyzed the correlations between birth weight and specific air pollutants, as well as maternal blood indicators that might be influenced by air pollutants. The primary objective of this study was to ascertain the associations between air pollution and maternal blood indicators, as well as fetal birth weight, within the groups of mothers with APS and those who were healthy controls. Our study contributes to the understanding of the potential health hazards posed by air pollution to pregnant women with APS and their fetuses. These findings will serve as a valuable scientific foundation for the formulation of public health policies. Implementing specific measures to enhance air quality and environmental conditions for pregnant women with APS can effectively mitigate health risks and promote overall maternal health during pregnancy.

## Materials and methods

### Study population

We conducted a pilot study at Nanjing Maternity and Child Health Care Hospital, Nanjing, China. APS pregnant women that were positive for at least one antiphospholipid antibody (anticardiolipin antibody, anti β2 glycoprotein-I, or lupus anticoagulant) were recruited. Healthy pregnant women without any prior medical conditions (e.g., diabetes, hypertension, and hepatitis B infection) were also recruited. After applying propensity score matching (1:1 matching) based on age and BMI, 189 healthy mothers were included in the control group. A total of 371 participants were considered eligible to participate in this study and thereby used for follow up analyses. Each participant signed an informed consent form voluntarily. The Nanjing Maternity and Child Health Care Hospital’s Institutional Review Board gave approval for the study.

### Assessment of exposure

#### PM_2.5_

Total mass of PM_2.5_ was expressed using the V5.GL.02 product of the Atmospheric Composition Analysis Group (ACAG) at Washington University in St. Louis (https://sites.wustl.edu/acag/datasets/surface-pm2-5/#V5.GL.02) (van Donkelaar et al. 2021). For each participant, we assessed monthly PM_2.5_ exposure by extracting concentrations at their geocoded residential address.

#### PM_10_, CO, NO_2_, and SO_2_

Daily concentrations of the four air pollutants were obtained from the National Environmental Monitoring Center of China (http://www.cnemc.cn/). There are 9 air pollution monitoring stations in Nanjing, Jiangsu Province, and 51 air pollution monitoring stations around Nanjing (Table S1). We used the inverse distance weighting (IDW) method to assess air pollutant exposure. For each participant, we calculated the predicted concentration at their home address as the inverse distance-weighted average of the concentrations at all neighboring monitoring stations.

#### O_3_

Ozone exposure dataset with a spatial resolution of 10 km was published by the China Air Pollution Tracking Organization (TAP, http://tapdata.org.cn/). For the specific methodology, we referred to a previous study (Xiao et al. 2022; Xue et al. 2020). For each participant, we assessed monthly mean ozone exposure by extracting concentrations at their geocoded residential address.

### Subject and sample collection

From January 2019 to June 2022, 371 blood samples were collected from 371 pregnant women at Nanjing Maternal and Child Health Care Hospital.

We collected late trimester blood from healthy pregnant women and pregnant women with antiphospholipid syndrome at 38 weeks gestation, and preterm pregnant women in both groups at 36 weeks. Blood routine, blood biochemistry and glycosylated hemoglobin data (including calcium (Ca), phosphorus (P), urea (BUN), creatinine (CREA), uric acid (UA), total protein (TP), albumin (ALB), total cholesterol (CHOL), triglyceride (TG), alanine aminotransferase (ALT), ASpartate aminotransferase (AST), alkaline phosphatase (ALP), lactate dehydrogenase (LDH), glutamine transpeptidase (GGT), high density lipoprotein (HDL), low density lipoprotein (LDL), total bilirubin (TBIL), direct bilirubin (DBIL), total bile acid (TBA), fasting blood) were detected Sugar (FBG), homocysteine (HCY), ferritin (SF), thyroid stimulating hormone (sTSH), anti-thyroid peroxidase antibody (anti-TPOAb), free thyroid hormone (FT4), glycated hemoglobin (HbA1C), white blood cells (WBC), red blood cells (RBC), hemoglobin (Hb), platelets (PLT), average platelet volume (MPV), platelet pressure (PCT), average red blood cell volume (MCV), average hemoglobin volume (MCH), average hemoglobin concentration (MCHC), neutrophil ratio (NEU_ratio), lymphocyte ratio (LYM_ratio), monocyte ratio (MON_ratio), eosinophilic ratio (EOS_ratio), basophilic ratio (BAS_ratio), neutrophil count (NEU), lymphocyte count (LYM), monocyte (MON), eosinophilic (EOS), basophilic (BAS), erythrocyte distribution width (RDW), RDW-CV, platelet distribution width (PDW), large platelet ratio (P_LCR), and hematocrit (HCT)). Blood routine was tested with the CAL8000 blood cell analyzer (Shenzhen, China), hemoglobin was tested with the ARKRAY HA-8180 hemoglobin analyzer (Japan), and blood biochemistry was tested with the BECKMAN AU5800 automatic biochemical analyzer (Miami, FL, USA).

Age at delivery, parity, gravidity, times of abortion, gestational hypertension, gestational diabetes, thyroid diseases, pre-pregnancy BMI, height, newborn gender, preterm birth, and relevant birth weights were extracted from medical records, and all data were averaged after two measurements of indicators.

### Statistical analysis

Statistical analyses were carried out in SPSS 25.0 (International Business Machines Corp., USA) or R-4.0.3 (R Development Core Team). The Kolmogorov-Smirnov test was used to analyze the measured data by the normality test. The standard deviation of normally distributed data was x ± standard deviation (SD). The t-test (variable conforming to normal distribution) or the Wilcoxon rank-sum test (variable not conforming to normal distribution) was performed to compare the two groups. Principal component analysis (PCA) was performed to explore the difference of blood indicator composition between healthy maternal and APS maternal by using package “factoextra” in R. Significant differences between healthy maternal and APS maternal blood indicator compositions were determined by permutational multivariate analysis of variance (PERMANOVA) and analysis of similarities (ANOSIM) by using R “vegan” package. The Mantel test was used to identify associations between blood indicators with APS by using R “vegan” package, and the greater the correlation of the Mantel test, the greater the strength of the potential effect of observed blood index on APS. Potential diagnostic biomarkers were assessed by receiver operating characteristic (ROC) curves by using R “pROC” package. The area under the ROC curve (AUC), a widely used metric for differentiation in risk prediction models, was reported and compared. The random forest (RF) analysis was used to identify the potential main blood indicator predictors of APS and healthy pregnant woman by using R “random forest” package (Jiao et al. 2018; Jin et al. 2017). Percentage increases in the mean squared error (MSE) was used to estimate the importance of these indicators, and higher MSE% values imply more important variables. Generalized linear model (GLM) was used to examine relationships of air pollutant components with different maternal blood indicators, and the relative importance for each of the predictors in this model was tested in nutshell and MASS package. Ridge regression was used to identify the associations between newborn birth weight with air pollutants, after eliminating the collinearity between each blood indicator and pollutants. Initially, univariate linear regression evaluated relationships between 16 distinctive blood biomarkers in late stage antiphospholipid syndrome-affected pregnant women and offspring body weight. Subsequently, biomarkers with significance below 0.05 underwent multivariate linear regression. This aimed to collectively gauge their impact within a comprehensive predictive model, elucidating significant factors and their interactions governing offspring weight.

## Results

### General characteristics of the study population

371 mothers were included in this study, 182 of whom were diagnosed with APS. Demographic characteristics of women were shown in Table 1. Maternal age, parity, gravidity, number of abortions, gestational hypertension, gestational diabetes mellitus, thyroid diseases during pregnancy, body mass index pre-pregnancy, height, weight pre-pregnancy, newborn gender, preterm birth, and low birth weight are shown in Table 1.

**Table 1:** Selected characters of mothers and newborns (*n* = 371). Data are presented as the mean ± standard deviation or number (%). **P < 0.01; ***P < 0.001

Characteristics	Mothers with non-APS	Mothers with APS	*P* value
	*N* (%)	*N* (%)	
Age (years)	30.82 ± 1.42	31.83 ± 3.69	0.66
Parity			
1	178.00 (94.18)	155.00 (85.16)	< 0.01**
2	11.00 (5.82)	25.00 (13.74)	0.01**
3	0.00 (0.00)	2.00 (1.10)	0.15
Gravidity			
1–2	174.00 (92.06)	102.00 (56.04)	< 0.001***
≥ 3	15.00 (7.94)	80.00 (43.96)	< 0.001***
Abortion ≥ 3 times	0.00 (0.0)	36.00 (19.78)	< 0.001***
Gestational hypertension	25.00 (13.23)	11.00 (6.04)	0.07
Gestational diabetes	38.00 (20.11)	51.00 (28.02)	0.07
Thyroid diseases	36.00 (19.05)	42.00 (23.08)	0.34
BMI (pre-pregnancy)	22.68 ± 6.42	21.73 ± 3.52	0.25
Height (mean ± SD)/cm	163.29 ± 4.12	161.79 ± 5.36	0.23
Weight (pre-pregnancy) (mean ± SD)/kg	56.53 ± 10.78	55.96 ± 6.90	0.58
Newborn gender			
Male	84.00 (44.44)	82.00 (45.05)	0.91
Female	105.00 (55.56)	90.00 (49.45)	0.24
Twins	0.00 (0.0)	10.00 (5.49)	< 0.001***
Preterm birth	5.00 (2.65)	13.00 (7.14)	0.07
Low birth weight	4.00 (2.12)	11.00 (6.04)	0.09

### Air pollution exposure assessments of pregnant women

The exposure concentrations of air pollutants are shown in Table 2. The mean concentrations of PM_2.5_, PM_10_, CO, NO_2_, SO_2_, and O_3_ reached Grade II level of the Chinese National Ambient Air Quality Standards (35 μg/m^3^, 70 μg/m^3^, 10 mg/m^3^, 40 μg/m^3^, 60 μg/m^3^, and 160 μg/m^3^, respectively) for each trimester during the study period.

**Table 2:** The air pollutant concentrations in different exposure windows (mean ± SD)

Exposure	Mothers with non-APS	Mothers with APS
	Mean ± SD	Mean ± SD
90 days before pregnancy		
PM_2.5_ (μg/m^3^)	54.47 ± 3.57	33.81 ± 9.48
PM_10_ (μg/m^3^)	19.98 ± 1.44	62.25 ± 14.47
CO (mg/m^3^)	1.22 ± 0.07	0.75 ± 0.07
NO_2_ (μg/m^3^)	54.35 ± 3.52	35.35 ± 7.41
SO_2_ (μg/m^3^)	19.98 ± 1.44	7.89 ± 1.37
O_3_ (μg/m^3^)	42.86 ± 6.22	118.29 ± 27.51
First trimester		
PM_2.5_ (μg/m^3^)	56.89 ± 2.42	33.33 ± 10.07
PM_10_ (μg/m^3^)	15.58 ± 0.922	61.21 ± 15.37
CO (mg/m^3^)	1.13 ± 0.04	0.74 ± 0.08
NO_2_ (μg/m^3^)	50.12 ± 2.74	33.94 ± 8.26
SO_2_ (μg/m^3^)	18.78 ± 1.01	7.36 ± 1.28
O_3_ (μg/m^3^)	83.67 ± 10.59	116.59 ± 28.95
Second trimester		
PM_2.5_ (μg/m^3^)	36.49 ± 9.14	39.63 ± 9.25
PM_10_ (μg/m^3^)	18.78 ± 1.01	59.79 ± 14.15
CO (mg/m^3^)	0.94 ± 0.05	0.71 ± 0.08
NO_2_ (μg/m^3^)	39.48 ± 3.61	33.27 ± 7.83
SO_2_ (μg/m^3^)	15.95 ± 0.92	6.83 ± 1.26
O_3_ (μg/m^3^)	131.89 ± 5.88	116.69 ± 25.16
Third trimester		
PM_2.5_ (μg/m^3^)	30.61 ± 2.68	31.69 ± 10.44
PM_10_ (μg/m^3^)	15.95 ± 0.92	58.54 ± 15.49
CO (mg/m^3^)	0.88 ± 0.06	0.71 ± 0.07
NO_2_ (μg/m^3^)	36.15 ± 3.37	32.97 ± 32.97
SO_2_ (μg/m^3^)	12.22 ± 1.38	6.61 ± 1.25
O_3_ (μg/m^3^)	103.89 ± 5.88	116.39 ± 25.49
Entire pregnancy		
PM_2.5_ (μg/m^3^)	44.62 ± 2.52	34.61 ± 3.91
PM_10_ (μg/m^3^)	12.22 ± 1.38	60.04 ± 5.93
CO (mg/m^3^)	0.98 ± 0.03	0.72 ± 0.04
NO_2_ (μg/m^3^)	41.85 ± 2.57	33.34 ± 3.39
SO_2_ (μg/m^3^)	15.58 ± 0.92	7.04 ± 0.98
O_3_ (μg/m^3^)	106.39 ± 4.84	117.12 ± 8.17

### APS and healthy maternal blood indicators variation

**Fig. 1:**
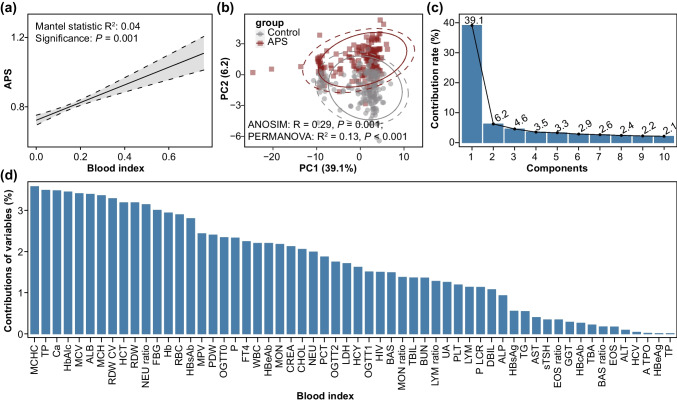
Analysis of blood indicators in APS versus healthy pregnant women. **a** The Mantel correlation test between maternal blood indicator compositions with maternal health status (with or without APS). **b** Principal component analysis (PCA) ordinations of maternal blood indicator composition between pregnant women with or without APS. **c** Contribution rate (%) of top ten components in PCA analysis. **d** Contributions (%) of different variables (blood indices) to the components of PC1 and PC2 in PCA analysis. ANOSIM, analysis of similarities; PERMANOVA, permutational multivariate analysis of variance

The correlation between maternal blood indicator compositions with maternal health status (with or without APS) was analyzed by the Mantel test, and the results noticed that the variation of maternal blood indicator compositions were significantly correlated with the occurrence of APS disease (Mantel test, *P* = 0.001; Fig. 1a). Furthermore, we also conducted principal component analysis (PCA) to visualize the differences in the composition of blood indicators from APS and healthy pregnant women (Fig. 1b). Base on the results of PERMANOVA and ANOSIM analyses, we noted that we were able to completely distinguish the blood indicator compositions of the APS group from the control group by PCA analysis (PERMANOVA, *P* < 0.001; ANOSIM, *P* = 0.001; Fig. 1b). Specifically, PCA results clearly showed that the blood indicator compositions from APS group were distinctly separated from control group almost along the second component (PC2). The cumulative contribution rate of each component (top ten were shown) was shown in Figure 1c, in which the first and second component (PC1 and PC2) explained 39.1% and 6.2% of the variability in the data of the blood indicators, respectively. In addition, we also calculated the combination contribution of each blood index to the PC1 and PC2 components, which was shown in the Figure 1d.

### Maternal blood indicators and APS disease association

**Fig. 2:**
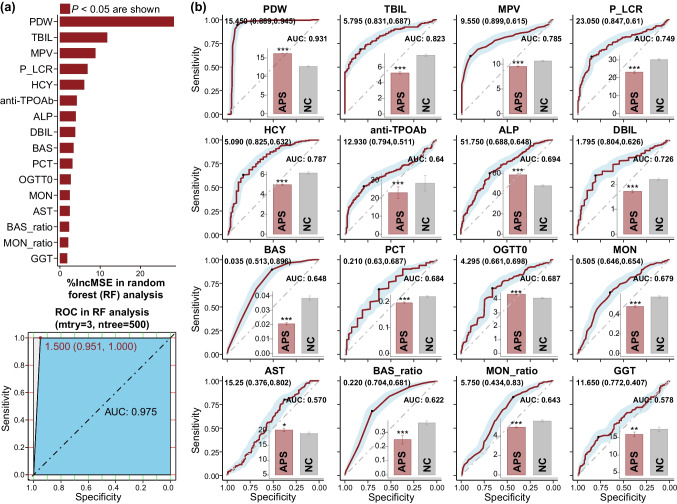
Prediction of APS by maternal blood indicators. **a** Random forest (RF) revealed the 16 most important blood indicators (*P* < 0.05) associated with the health status of pregnant women (with or without APS) are shown. Receiver operating characteristic curve (ROC) in the RF analysis. **b** ROC curve of each blood indicator and bar plots illustrate the distribution of blood indicators in the healthy pregnant women group (NC, gray) versus the APS pregnant women group (APS, red). Asterisks indicate significant differences between the healthy pregnant women group (NC) and the APS pregnant women group (APS) as determined by the Wilcoxon test (^*^, ^**^, and ^***^ for *P* < 0.05, *P* < 0.01, and *P* < 0.001, respectively)

We used random forest (RF) analysis to disentangle the potential main predictors in maternal blood that associated with the health status of pregnant women (with or without APS). We divided the data set into training set (70% of the total data) and test set (30% of the total data), and then we modeled using the training set and verified using the test set. The receiver operating characteristic curve (ROC) and the area under curve (AUC) score were used to verify the accuracy rate of RF model to predict the classification of samples. We noticed that an AUC (0.975) greater than 0.9 was found in our RF analysis, which was considered to have the ability to diagnose APS with great specificity and sensitivity (Fig 2a). Specifically, the random forest models resulted in the selection of a total of sixteen blood indicators as the main blood predictors that contribute to APS disease (*P* < 0.05). PDW, which was enriched in APS group (Wilcoxon test, *P* < 0.001), was found to be the most important variable for predicting APS disease occurrence followed by TBIL, MPV, P_LCR, HCY, anti-TPOAb, ALP, DBIL, BAS, PCT, OGTT0, MON, AST, BAS_ratio, MON_ratio, and GGT. In addition, Wilcoxon test analyses indicated that significant difference of these remaining fifteen blood indicators were also observed as compared healthy pregnant women group versus APS pregnant women group (*P* < 0.05; Fig. 2b).

ROC analysis was also used to verify the diagnostic validity of these sixteen blood indicators to APS disease occurrence, and an AUC greater than 0.7 was considered to have the ability to diagnose APS with great specificity and sensitivity (Fig. 2b). ROC analysis results showed that the AUCs of blood indicators of PDW, TBIL, MPV, P_LCR, HCY, and DBIL were 0.931, 0.823, 0.785, 0.749, 0.787, and 0.726, respectively, suggesting these blood indicators can be used to predict APS disease occurrence and with good specificity and sensitivity.

### Association of air pollution and maternal blood indicators

**Fig. 3:**
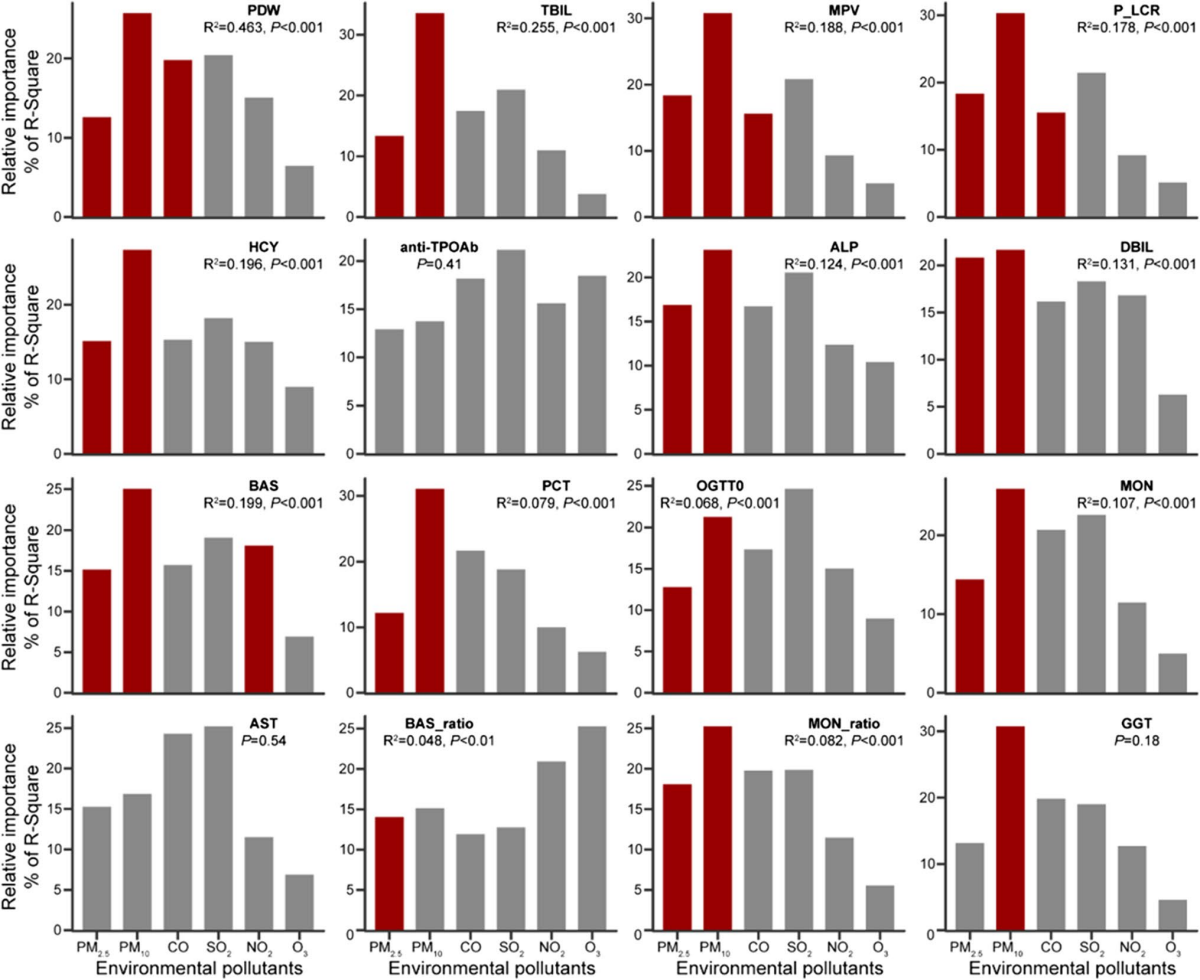
Effects of atmospheric pollutants on maternal blood indicators. Generalized linear model (GLM) analysis revealed the magnitude of the effects for different atmospheric pollutants on maternal blood indicators. Bars with red color represent the atmospheric pollutants that significantly affect the selected blood indicator (*P* < 0.05). Model is adjusted for possible confounding variables, including age at delivery, parity, gravidity, times of abortion, gestational hypertension, gestational diabetes, thyroid diseases, pre-pregnancy BMI, height, newborn gender, and preterm birth

To further investigate the atmospheric pollutants that significantly affect these sixteen important maternal blood indicators, we used generalized linear model (GLM) analysis to examine the relationships of different atmospheric pollutants with maternal blood indicators and identify the relative importance for each of the predictors (Fig. 3). GLM analysis indicated that the atmospheric pollutants of PM_2.5_ and PM_10_ were the most important contributors (*P* < 0.05) to these twelve maternal blood indicators comprised of PDW, TBIL, MPV, P_LCR, HCY, ALP, DBIL, BAS, PCT, OGTT0, MON, and MON_ratio. In addition, the atmospheric pollutant CO was the important contributor (*P* < 0.05) of these three blood indicators comprised of PDW, MPV, and P_LCR. However, all atmospheric pollutants did not significantly affect the blood indicators of anti-TPOAb and AST.

### Potential effects of prenatal air pollution exposure on birth weight

**Fig. 4:**
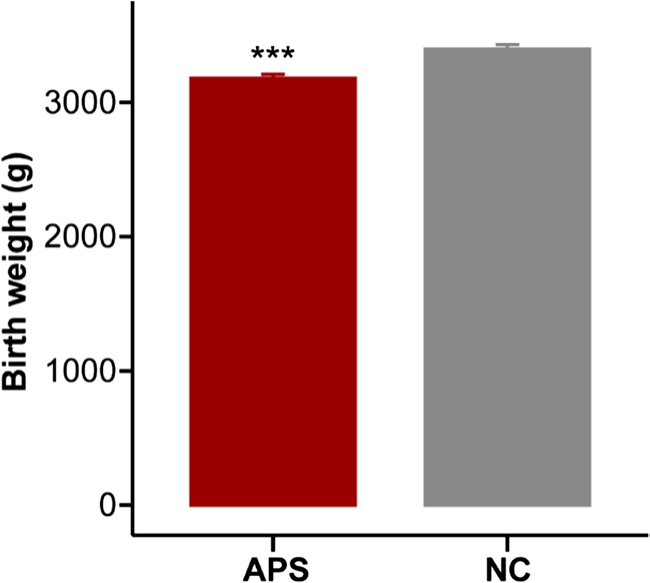
Birth weight of the offspring of APS mothers versus healthy mothers. Birth weight of the healthy pregnant women group (NC, gray) versus the APS pregnant women group (APS, red). Asterisks indicate significant differences between NC group and APS group as determined by the Wilcoxon test (^***^P < 0.001)

The Wilcoxon test showed that birth weight was significantly lower in the APS group (APS) compared to the control group (NC) (Fig. 4). Using a ridge regression model, we further examined the relationship between birth weight and air contaminants according to various exposure periods. Birth weight was statistically linked with maternal PM_2.5_, PM_10_, and SO_2_ exposures, as indicated in Table 3. We found a negative correlation between birth weight and PM_2.5_ exposure in mid- and late pregnancy, respectively, after stratifying by gestation (*P* < 0.05, respectively). But in the first 90 days of pregnancy, early and mid-trimester SO_2_ exposure were found to be positively correlated with birth weight (*P* < 0.05, respectively).

**Table 3:** The relationship between birth weight and prenatal exposure to air pollutants

Characters	Pollutants	Entire pregnancy		90 days before pregnancy		First trimester		Second trimester		Third trimester	
		***β***	***P***	***β***	***P***	***β***	***P***	***β***	***P***	***β***	***P***
Weight		*k*^a^ = 0.159		*k*^a^ = 0.136		*k*^a^ = 0.107		*k*^a^= 0.128		*k*^a^ = 0.129	
	PM_2.5_	−0.373	**< 0.001**	−0.014	0.815	−0.048	0.456	−0.496	**< 0.001**	−0.030	**0.014**
	PM_10_	−0.169	**< 0.001**	−0.067	0.217	−0.060	0.296	−0.018	0.707	−0.010	0.194
	CO	0.092	0.332	0.042	0.415	0.029	0.632	0.050	0.299	0.016	0.245
	NO_2_	0.073	0.180	−0.042	0.499	0.078	0.252	0.018	0.702	0.011	0.409
	SO_2_	0.192	**< 0.001**	0.130	**0.010**	0.128	**0.025**	0.091	**0.041**	0.022	0.051
	O_3_	−0.073	0.451	−0.023	0.023	−0.010	0.897	−0.025	0.588	−0.018	0.132

The significance of bold is defined as that the *P* value is less than 0.05. *k*^a^ was the ridge parameter. The model is adjusted for possible confounding variables, including times of abortion, gestational hypertension, gestational diabetes, thyroid diseases, pre-pregnancy BMI, height, newborn gender, and preterm birth.

### Potential effects of APS and air pollution-related maternal blood indicators on birth weight

We further examined the relationships between birth weight and APS and air pollution-related maternal blood indicators with differential variations, as shown in Table 4. In both univariate and multivariate analyses, among the maternal blood indicators related to APS and air pollution, we noted that only the blood PDW level and MON level of pregnant women in the third trimester were significantly negatively correlated with birth weight (*P* < 0.05).

**Table 4:** Associations between APS and air pollution-related maternal blood indicators with birth weight

Characters	Blood indicators	Univariate analysis		Multivariate analysis	
		95% CI	*P* value	95% CI	*P* value
Weight	OGTT0	4.19–4.31	0.037	–	–
	anti-TPOAb	20.13–29.37	0.241	–	–
	BAS	0.02–0.03	0.029	–	–
	PCT	0.20–0.21	0.023	–	–
	BAS ratio	0.28–0.35	0.450	–	–
	**MON**	**0.51–0.54**	**< 0.001**	**0.50–0.56**	**<0.05**
	MON ratio	5.13–5.39	0.022	–	–
	MPV	9.95–10.25	0.326	–	–
	**PDW**	**14.06–14.56**	**< 0.001**	**14.13–14.68**	**< 0.001**
	P_LCR	25.85–27.75	0.370	–	–
	DBIL	1.89–2.04	0.475	–	–
	HCY	5.41–5.69	0.096	–	–
	TBIL	6.17–6.66	0.140	–	–
	GGT	15.76–17.68	0.248	–	–
	AST	18.83–20.40	0.437	–	–
	ALP	50.85–53.86	0.035	–	–

The significance of bold is defined as that the *P* value is less than 0.05.

Multivariate analysis model is adjusted for possible confounding variables, including times of abortion, gestational hypertension, gestational diabetes, thyroid diseases, pre-pregnancy BMI, height, and preterm birth.

### Discussion

The occurrence of human diseases is often accompanied by extensive changes in blood indicators (Bath et al. 2004). Similarly, we also found PDW, TBIL, MPV, P_LCR, HCY, and DBIL to be better predictors of pregnant women APS disease occurrence by RF and ROC analysis. Platelet size (MPV, PDW, and P_LCR) is considered as a standard indicator of platelet function in disease pathophysiology (Asare et al. 2019). The patient’s prethrombotic condition may change as a result of changes in platelet parameters. Elevated MPV levels, which have been found to be a risk factor for myocardial infarction and stroke, are one of the clinical disorders that platelet markers have been linked to (Bath et al. 2004; Huczek et al. 2005). And there is evidence of a correlation between PDW and thrombotic events in patients with APS (Shi et al. 2021), and our findings suggest that significant changes in platelet-related markers (PDW, MPV, P_LCR) in APS mothers in late pregnancy compared to controls may be associated with the risk of thrombosis. We did not find any studies on TBIL versus DBIL in APS mothers, but it has been documented that bilirubin is able to bind to fibrinogen involved in coagulation and prevent the oxidation of fibrinogen, and oxidatively modified fibrinogen in vivo and in vitro is more likely to be involved in thrombosis (Becatti et al. 2014; Hugenholtz et al. 2016). Bilirubin is an endogenous anti-inflammatory marker associated with thrombosis in studies of patients with atherosclerosis and lower bilirubin levels in venous thromboembolism (VTE) (Duman et al. 2019). This is consistent with our study. Hyperhomocysteinemia has been associated with a variety of obstetric issues, including pre-eclampsia, placental abruption, fetal death, and recurrent spontaneous abortion (Nelen et al. 2000a; Nelen et al. 2000b; Unfried et al. 2002). However, it has also been suggested that hyperhomocysteinemia is not associated with APS, and that elevated HCY may be an independent risk factor for thrombosis (Lee et al. 2004), so it is not possible to clarify the consequences caused by reduced HCY in late pregnancy in APS mothers in this study for the time being.

There is concern about the impact of air pollution on maternal health owing to increased awareness about exposure to environmental toxicants may contribute to the pathogenesis of pregnancy diseases. In the present study, we found that PM_2.5_ and PM_10_ were the most important variables affecting multiple maternal blood markers. Similar results were also observed in previous studies that chronic exposure to higher levels of air pollutants (PM_1_, PM_2.5_, PM_10_, and NO_2_) was associated with increased platelet size (Hou et al. 2020), and in addition to systemic inflammation (Ferrucci and Fabbri 2018), the pro-thrombotic pathway induced by particulate PM may also be an important biological pathway contributing to cardiovascular risk (Brook and Rajagopalan 2010; Dehghani et al. 2022b; Rajagopalan et al. 2018). Results from rodent model studies suggest that exposure to airborne PM may lead to platelet aggregation and arterial thrombosis (Hadei and Naddafi 2020). Epidemiological studies have shown that exposure to high concentrations of PM_2.5_ is associated with reduced MPV, increased PDW, and higher platelet counts (Viehmann et al. 2015; Yin et al. 2017; Zhang et al. 2018). This is consistent with our findings which suggests that PM_2.5_ and PM_10_ may cause further changes in clinical biomarkers associated with maternal platelets in APS. The location of deposition of PM is mainly determined by particle size. For example, coarse particulate matter is usually deposited in the upper airways and larger airways, whereas ultrafine particulate matter can penetrate into the body circulation and subsequently directly affect remote organs or tissues such as the liver and adipose tissue (Peng et al. 2008; Rajagopalan et al. 2018). The liver function items TBIL and DBIL were positively correlated with PM_2.5_ concentrations (Deng et al. 2020; Zhang et al. 2021), and there was an association between greater PM exposure and elevated HCY (Yang et al. 2020). In the present study, APS mothers showed a tendency to have lower TBIL, DBIL, and HCY in late pregnancy compared to normal mothers, a result that may be due to the specificity of the study population, the different PM concentrations and exposure windows, and the fact that the study population was treated to varying degrees during pregnancy. Therefore, further well-designed studies and animal studies are needed to obtain more accurate information on whether PM can affect maternal liver function parameters in APS. Our results provide evidence for the effect of exposure to atmospheric pollutants during pregnancy on maternal blood markers associated with APS in late pregnancy.

Some studies have shown that urban pollution exposure during pregnancy triggers fetal growth restriction (Dehghani et al. 2022a; Slama et al. 2009), low birth weight (Shah et al. 2011), and preterm birth (Zhao et al. 2011). However, the findings are inconsistent. In our analysis, exposure to PM_2.5_, PM_10_, and SO_2_ throughout pregnancy was associated with birth weight. After stratifying by gestational period, exposure to high PM_2.5_ in mid- and late pregnancy was found to cause lower birth weight, and our results are consistent with previous studies on the negative association between PM_2.5_ exposure and birth weight (Pedersen et al. 2013; Stieb et al. 2016; Yitshak-Sade et al. 2021). According to a meta-analysis, each 10 μg/m^3^ rise in PM_10_ was associated with an increased risk of low birth weight throughout pregnancy as well as in the first, second, and third trimesters (Feng et al. 2017). And in this study, no effect of PM_10_ exposure on birth weight was observed after stratification by trimester. In conclusion, regional differences in PM concentration, composition, and size may be the primary cause of the discrepancies between the findings of earlier investigations. In terms of SO_2_, our research revealed a strong positive correlation between birth weight and SO_2_ exposure at various exposure windows. However, most studies reported a statistically significant association between SO_2_ exposure and lower birth weight (Jacobs et al. 2017), and differences between studies may be due to differences in pollutant concentration, exposure duration, and pollutant misclassification. A significant factor may also be the fact that covariance between various pollutants was not taken into account in earlier investigations. Another important reason is that due to objective reasons, we can only investigate the sulfur dioxide situation in the residence of pregnant women, and the sulfur dioxide content and exposure time of pregnant women in their daily life cannot be known. We need to expand the sample size and conduct in-depth investigation on the living conditions of pregnant women before conducting research. Since prenatal exposure to air pollutants may lead to birth weight loss (Dehghani et al. 2022a) through placental transfer and oxidative stress, there is still little research on the mechanism of SO_2_-induced fetal weight change, whether the concentration range of SO_2_ in this study can cause maternal oxidative stress response and changes in blood inflammatory factors. And transmission to the fetus still need further experimental proof.

Notably, we found a significant negative correlation between PDW and MON and birth weight (*P* < 0.05); nevertheless, we could not find any study on the correlation between maternal PDW and MON and birth weight, and to our knowledge, our study is the first reported study, but further studies are needed to prove whether PDW can further predict lower birth weight.

This study is the first to explore the effects of atmospheric pollutants on differential maternal blood indicators in late pregnancy in APS mother group, but some limitations remain: our assessment of environmental exposure was limited to the location of the residential address and did not record the daily activities of each pregnant woman, so our assessment of maternal environmental exposure to atmospheric pollutants may be misclassified. In addition, this study was a single-center study and may have had limitations on the population investigated. And due to objective factors, potential confounders such as information on the duration or timing of air pollution exposure during pregnancy, maternal smoking history, diet during pregnancy, or socioeconomic status could not be obtained in this study. Moreover, this study only investigated the association between a limited number of pollutants and blood indicators, and in future studies, we will investigate the association between other pollutants and blood indicators in pregnant women with antiphospholipid syndrome. Through this study, we found that air pollution is correlated with the blood indexes and offspring weight of pregnant women with antiphospholipid syndrome. Therefore, the different effects of air pollution, different components, different exposure window periods, exposure time, and absorbed dose on the weight of pregnant women with antiphospholipid syndrome and offspring may be the direction of future research.

## Conclusion

In conclusion, this study provides the first human evidence of significant differences in 16 blood markers in late pregnancy between healthy mothers and mothers with APS, with PDW being a strong predictor of APS. In this study, PM_2.5_ and PM_10_ had the greatest impact on 12 different blood indexes such as PDW. Birth weight was negatively associated with PM_2.5_ exposure in the second and third trimesters, respectively. The levels of blood PDW and MON in the third trimester were negatively correlated with the birth weight. These findings suggest that differences in blood indicators in late pregnancy between healthy mothers and APS mothers, as well as birth weight, are associated with maternal environmental exposure during pregnancy. They provide the fundamental data necessary for further studies to assess the health effects of atmospheric environmental exposure using biomarkers. This study will help us better design clinical or animal experiments to further confirm the effects and mechanisms of air pollution on APS mothers and their offspring.

### Supplementary Information

ESM 1 (DOCX 21.6 KB)

## Data Availability

All data generated or analyzed during this study are included and available in this article.

## Abbreviations

APS: Antiphospholipid syndrome
PM: Particulate matter
PM_2.5_: Fine particulate matter 2.5
PM_10_: Particulate matter 10
SO_2_: Sulfur dioxide
NO_2_: Nitrogen dioxide
CO: Carbon monoxide
O_3_: Ozone
PDW: Platelet distribution width
MPV: Mean platelet volume
P_LCR: Platelet-larger cell ratio
TBIL: Total bilirubin
DBIL: Direct bilirubin
HCY: Homocysteine
Anti-TPOAb: Antithyroid peroxidase autoantibody
ALP: Alkaline phosphatase
BAS: Basophilic granulocyte
BAS ratio: Basophilic granulocyte ratio
PCT: Platelet thrombocytocrit
OGTT0: Preprandial glucose levels
MON: Monocytes
MON ratio: Monocytes ratio
AST: Aspartate aminotransferase
GGT: Glutamyl transpeptidase
