# Supplementary material for: Association between ambient air pollution exposure in pregnant women with antiphospholipid syndrome in Nanjing, China
Source: Environ Sci Pollut Res Int. 2023 Nov 1;30(54):116266–78. doi: 10.1007/s11356-023-29937-0 (PMC10682106; doi:10.1007/s11356-023-29937-0)
Supplement: Supplementary file 1 — (DOCX 21.6 KB) [file 11356_2023_29937_MOESM1_ESM.docx]

*Environmental Science and Pollution Research*

**Association between ambient air pollution exposure in pregnant women with antiphospholipid syndrome in Nanjing, China**

Bimei Hu^b,1^, Linjie Xu^a,1^, Xu Yang ^d,1^, Shiwen Qu^b^, Lan Wu^a^, Yumei Sun^c^, Jun Yan^a^, Yexiao Zhang^a^, Zhaoer Yu^a^, Yixiao Wang^a^, Ruizhe Jia^a,^*

* Corresponding author: Ruizhe Jia, Department of Obstetrics, Women’s Hospital of Nanjing Medical University, Nanjing Maternity and Child Health Care Hospital, 210000, Nanjing, China. E-mail: jiaruizhe2016@163.com.

**Supplemental information**

**Table S1. Air pollution monitoring stations in and around Nanjing.**

| Number of air pollution monitoring stations | City | Longitude | Latitude |
| --- | --- | --- | --- |
| X1151A | Nanjing | 118.80 | 32.11 |
| X1152A | Nanjing | 118.75 | 32.06 |
| X1153A | Nanjing | 118.78 | 32.07 |
| X1154A | Nanjing | 118.78 | 32.01 |
| X1155A | Nanjing | 118.80 | 32.03 |
| X1156A | Nanjing | 118.80 | 32.08 |
| X1157A | Nanjing | 118.63 | 32.09 |
| X1158A | Nanjing | 118.74 | 32.01 |
| X1159A | Nanjing | 118.91 | 32.11 |
| X1798A | Ma'anshan | 118.51 | 31.69 |
| X1799A | Ma'anshan | 118.48 | 31.64 |
| X1800A | Ma'anshan | 118.51 | 31.75 |
| X1801A | Ma'anshan | 118.48 | 31.69 |
| X1802A | Ma'anshan | 118.64 | 31.71 |
| X2298A | Chuzhou | 118.31 | 32.32 |
| X2299A | Chuzhou | 118.32 | 32.28 |
| X2300A | Chuzhou | 118.32 | 32.31 |
| X1184A | Yangzhou | 119.46 | 32.39 |
| X1185A | Yangzhou | 119.40 | 32.41 |
| X1186A | Yangzhou | 119.39 | 32.38 |
| X1187A | Yangzhou | 119.44 | 32.40 |
| X1203A | Zhenjiang | 119.68 | 32.19 |
| X1204A | Zhenjiang | 119.49 | 32.22 |
| X1205A | Zhenjiang | 119.43 | 32.13 |
| X1196A | Changzhou | 120.00 | 31.76 |
| X1197A | Changzhou | 119.93 | 31.78 |
| X1198A | Changzhou | 119.96 | 31.81 |
| X1199A | Changzhou | 120.04 | 31.76 |
| X1200A | Changzhou | 119.94 | 31.70 |
| X1201A | Changzhou | 119.91 | 31.91 |
| X1794A | Wuhu | 118.35 | 31.35 |
| X1795A | Wuhu | 118.37 | 31.42 |
| X1796A | Wuhu | 118.37 | 31.32 |
| X1797A | Wuhu | 118.40 | 31.38 |
| X2316A | Xuancheng | 118.76 | 30.94 |
| X2317A | Xuancheng | 118.74 | 30.97 |
| X2318A | Xuancheng | 118.72 | 30.94 |
| X1188A | Wuxi | 120.27 | 31.49 |
| X1189A | Wuxi | 120.28 | 31.62 |
| X1190A | Wuxi | 120.29 | 31.56 |
| X1191A | Wuxi | 120.24 | 31.50 |
| X1192A | Wuxi | 120.35 | 31.58 |
| X1193A | Wuxi | 120.35 | 31.55 |
| X1194A | Wuxi | 120.25 | 31.56 |
| X1195A | Wuxi | 120.29 | 31.68 |
| X2270A | Bengbu | 117.40 | 32.94 |
| X2271A | Bengbu | 117.36 | 32.94 |
| X2272A | Bengbu | 117.31 | 32.94 |
| X2273A | Bengbu | 117.42 | 32.89 |
| X2274A | Bengbu | 117.35 | 32.97 |
| X2275A | Bengbu | 117.31 | 32.90 |
| X2285A | Tongling | 117.82 | 30.94 |
| X2286A | Tongling | 117.81 | 30.92 |
| X2287A | Tongling | 117.78 | 30.94 |
| X2288A | Tongling | 117.74 | 30.88 |
| X2289A | Tongling | 117.86 | 30.92 |
| X2290A | Tongling | 117.85 | 30.97 |
| X1249A | Huzhou | 120.10 | 30.89 |
| X1250A | Huzhou | 120.07 | 30.82 |
| X1251A | Huzhou | 120.09 | 30.86 |
